# Potent Neutralization of SARS-CoV-2 by Hetero-Bivalent Alpaca Nanobodies Targeting the Spike Receptor-Binding Domain

**DOI:** 10.1128/JVI.02438-20

**Published:** 2021-04-26

**Authors:** Huan Ma, Weihong Zeng, Xiangzhi Meng, Xiaoxue Huang, Yunru Yang, Dan Zhao, Peigen Zhou, Xiaofang Wang, Changcheng Zhao, Yong Sun, Peihui Wang, Huichao Ou, Xiaowen Hu, Yan Xiang, Tengchuan Jin

**Affiliations:** a Department of Pulmonary and Critical Care Medicine, The First Affiliated Hospital of USTC, Division of Life Sciences and Medicine, University of Science and Technology of China, Hefei, Anhui, China; b Hefei National Laboratory for Physical Sciences at Microscale, Laboratory of Structural Immunology, CAS Key Laboratory of Innate Immunity and Chronic Disease, Division of Life Sciences and Medicine, University of Science and Technology of China, Hefei, Anhui, China; c Department of Microbiology, Immunology, and Molecular Genetics, University of Texas Health Science Center at San Antonio, San Antonio, Texas, USA; d Department of Statistics, University of Wisconsin—Madison, Madison, Wisconsin, USA; e The First Affiliated Hospital of USTC, Division of Life Sciences and Medicine, University of Science and Technology of China, Hefei, Anhui, China; f Department of Infectious Diseases, The First Affiliated Hospital of USTC, Division of Life Sciences and Medicine, University of Science and Technology of China, Hefei, Anhui, China; g Anhui Provincial Center for Disease Control and Prevention, Hefei, Anhui, China; h Key Laboratory for Experimental Teratology of Ministry of Education and Advanced Medical Research Institute, Cheeloo College of Medicine, Shandong University, Jinan, China; i CAS Center for Excellence in Molecular Cell Science, Chinese Academy of Science, Shanghai, China; Loyola University Chicago

**Keywords:** SARS-CoV-2, COVID-19, nanobody, antibody, alpaca, hetero-bivalent

## Abstract

Cellular entry of SARS-CoV-2 requires the binding between the receptor-binding domain (RBD) of the viral Spike protein and the cellular angiotensin-converting enzyme 2 (ACE2). As such, RBD has become the major target for vaccine development, whereas RBD-specific antibodies are pursued as therapeutics. Here, we report the development and characterization of SARS-CoV-2 RBD-specific V_H_H/nanobody (Nb) from immunized alpacas. Seven RBD-specific Nbs with high stability were identified using phage display. They bind to SARS-CoV-2 RBD with affinity *K_D_* ranging from 2.6 to 113 nM, and six of them can block RBD-ACE2 interaction. The fusion of the Nbs with IgG1 Fc resulted in homodimers with greatly improved RBD-binding affinities (*K_D_* ranging from 72.7 pM to 4.5 nM) and nanomolar RBD-ACE2 blocking abilities. Furthermore, the fusion of two Nbs with nonoverlapping epitopes resulted in hetero-bivalent Nbs, namely, aRBD-2-5 and aRBD-2-7, with significantly higher RBD binding affinities (*K_D_* of 59.2 pM and 0.25 nM) and greatly enhanced SARS-CoV-2 neutralizing potency. The 50% neutralization doses of aRBD-2-5 and aRBD-2-7 were 1.22 ng/ml (∼0.043 nM) and 3.18 ng/ml (∼0.111 nM), respectively. These high-affinity SARS-CoV-2 blocking Nbs could be further developed into therapeutics, as well as diagnostic reagents for COVID-19.

**IMPORTANCE** To date, SARS-CoV-2 has caused tremendous loss of human life and economic output worldwide. Although a few COVID-19 vaccines have been approved in several countries, the development of effective therapeutics, including SARS-CoV-2 targeting antibodies, remains critical. Due to their small size (13 to 15 kDa), high solubility, and stability, Nbs are particularly well suited for pulmonary delivery and more amenable to engineer into multivalent formats than the conventional antibody. Here, we report a series of new anti-SARS-CoV-2 Nbs isolated from immunized alpaca and two engineered hetero-bivalent Nbs. These potent neutralizing Nbs showed promise as potential therapeutics against COVID-19.

## Abstract (precis)

To date, SARS-CoV-2 has caused tremendous loss of human life and economic output worldwide. Although a few COVID-19 vaccines have been approved in several countries, the development of effective therapeutics, including SARS-CoV-2 targeting antibodies, remains critical.

## INTRODUCTION

Coronavirus disease 2019 (COVID-19) caused by SARS-CoV-2 has resulted in tremendous lives and economic losses worldwide. SARS-CoV-2 belongs to the betacoronavirus genus, which includes two other significant human pathogens, severe acute respiratory syndrome coronavirus (SARS-CoV-1) and Middle East respiratory syndrome coronavirus (MERS-CoV), that first emerged in humans in 2002 and 2012, respectively (1–4). Currently, several COVID-19 vaccines have been approved for emergency usages by several countries (5, 6). Remdesivir (7) and dexamethasone (8) have also been approved for treating COVID-19 under emergency use authorization. However, to more effectively combat COVID-19 and prepare for possible future pandemics, it remains essential to develop new drugs targeting coronaviruses.

Virus-specific antibody responses can be readily detected in sera of COVID-19 patients (9–12), and a series of monoclonal antibodies (MAbs) that neutralize SARS-CoV-2 have been isolated from infected individuals (13–18). Both convalescent plasma and MAbs targeting SARS-CoV-2 have shown promise as therapeutics for treating COVID-19 patients (19–21). In addition to the conventional MAbs, a distinct antibody fragment derived from camelid immunoglobulins, termed V_H_H or nanobody (Nb), is an attractive alternative for COVID-19 treatment. Compared to the conventional antibody, V_H_H is less expensive to produce, has an enhanced tissue penetration, and is more amenable to engineering into multivalent and multispecific antigen-binding formats (22). Moreover, Nbs are particularly well suited for pulmonary delivery because of their small size (13 to 15 kDa), high solubility, and stability (23, 24).

Cell entry by SARS-CoV-2 requires the interaction between the receptor-binding domain (RBD) of the viral Spike protein and the cellular angiotensin-converting enzyme 2 (ACE2), which is also the receptor for SARS-CoV-1 (25–29). The RBD of SARS-CoV-2 binds to ACE2 about 10- to 20-fold better than that for SARS-CoV-1 RBD in some studies (30).

This study reports the development and characterization of seven anti-RBD Nbs isolated from alpacas immunized with SARS-CoV-2 RBD. Furthermore, two high-affinity hetero-bivalent Nbs were developed by fusing two Nbs with distinct epitopes, resulting in antibodies with strong SARS-CoV-2 neutralizing potency.

## RESULTS

### Highly stable anti-SARS-CoV-2 RBD nanobodies were isolated from immunized alpacas.

**FIG 1:**
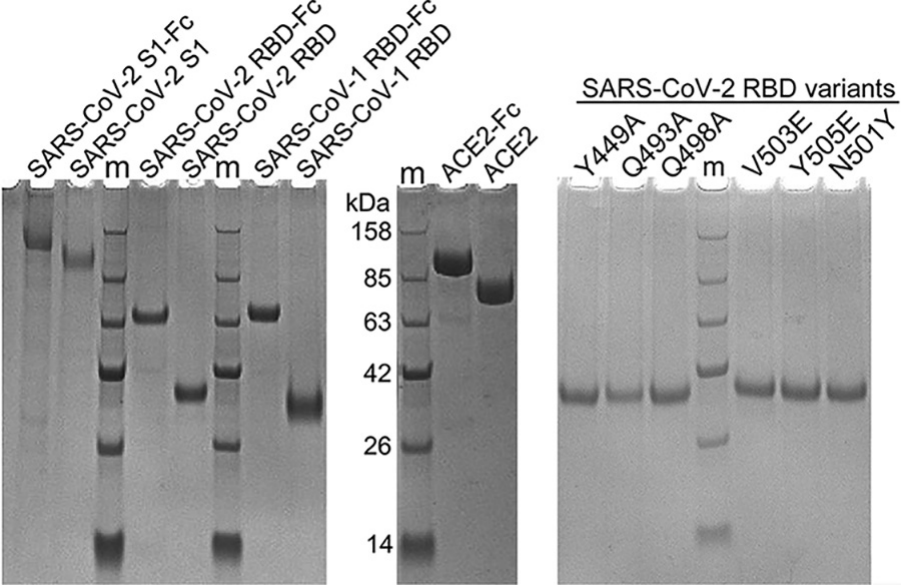
Reduced SDS-PAGE analysis of the purified proteins used in this study. All the proteins were expressed as fusions with a TEV protease cleavage site and the human IgG1 Fc in HEK293F cells. The fusions were purified from culture supernatants with protein A, while the proteins without TEV-Fc were obtained from the flowthrough of protein A and Ni NTA after digestion with the TEV protease. “m” indicates a protein ladder.

**FIG 2:**
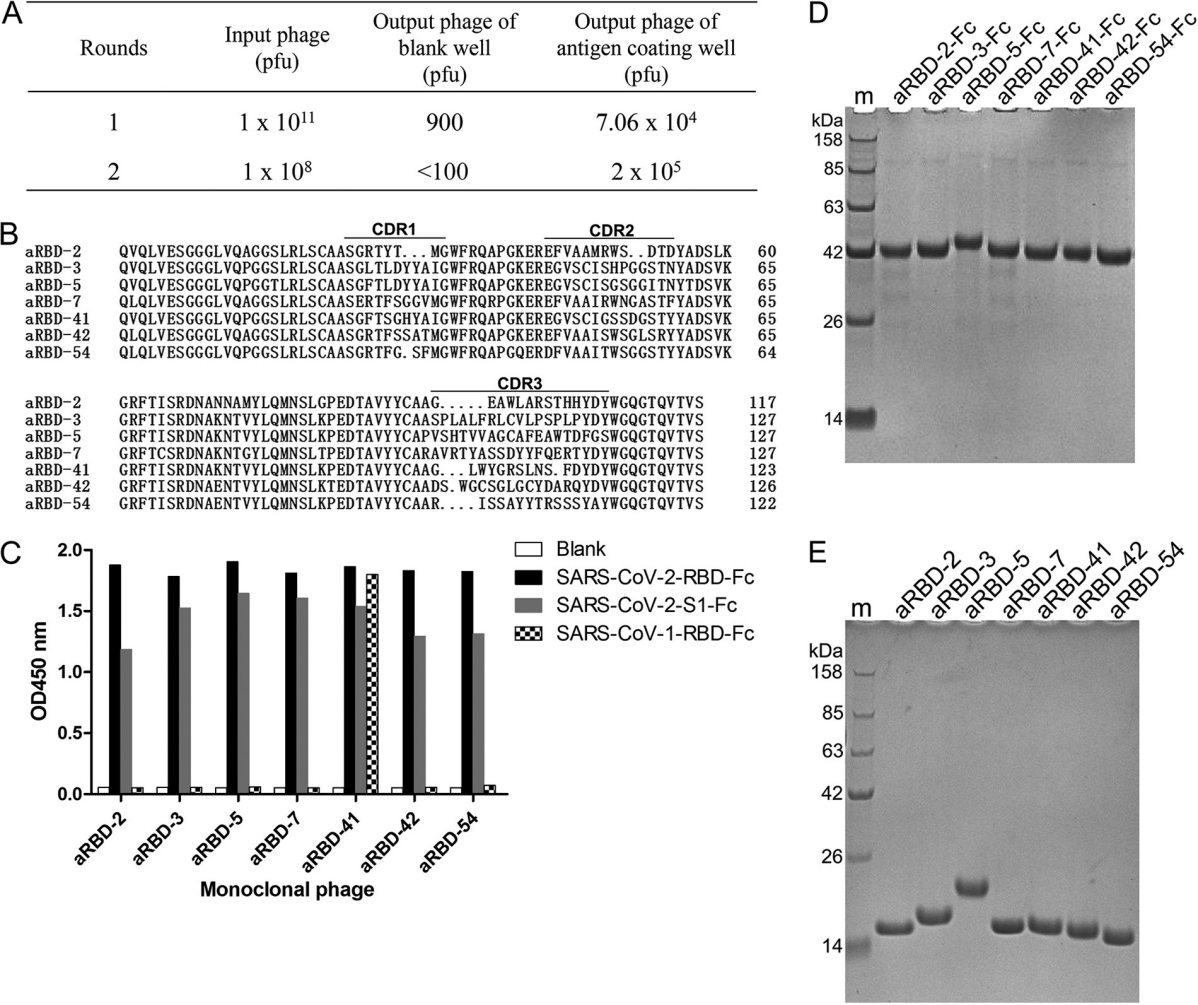
Isolation of anti-SARS-CoV-2 RBD Nbs from phage display library derived from RBD-immunized alpacas. (A) Enrichment of phages after panning on SARS-CoV-2 RBD. (B) The amino acid sequence of the isolated seven anti-RBD Nbs. (C) ELISA results for characterizing the binding of the isolated seven Nbs to SARS-CoV-2 RBD-Fc, SARS-CoV-2 S1-Fc and SARS-CoV-1 RBD-Fc. (D) Reduced SDS-PAGE analysis of the purified Nb-Fc fusions. (E) Reduced SDS-PAGE analysis of the purified Nbs.

We aimed to develop potent SARS-CoV-2 neutralizing antibodies with favorable biological characteristics. Towards this goal, we immunized two alpacas three times with highly purified recombinant SARS-CoV-2 RBD (Fig. 1). Total RNA was extracted from 1 × 10^7^ peripheral blood mononuclear cells from the immunized alpacas and used as the template for synthesizing cDNA. The V_H_H coding regions were amplified from the cDNA and cloned into a phagemid vector, generating a library with about 1.6 × 10^7^ independent clones. Phages displaying V_H_H were prepared from the library with the helper phage and selected with SARS-CoV-2 RBD via two rounds of biopanning. After each round of panning, titration of the output phages indicated that the RBD-binding phages were effectively enriched (Fig. 2A). Individual phages were also randomly picked, and their RBD-binding activity was evaluated with phage enzyme-linked immunosorbent assay (ELISA). Of 31 phages analyzed, 19 and 30 respectively, were found to be positive for RBD binding after the first and second rounds of panning (data not shown), again indicating the enrichment of RBD-binding phages.

After two rounds of panning, sequencing of the positive phage clones revealed seven unique Nbs (Fig. 2B), which were named aRBD-2, aRBD-3, aRBD-5, aRBD-7, aRBD-41, aRBD-42, and aRBD-54. All seven phages can bind to SARS-CoV-2 RBD and the S1 domain in ELISA, and one (aRBD-41) can also bind to SARS-CoV-1 RBD (Fig. 2C).

**FIG 3:**
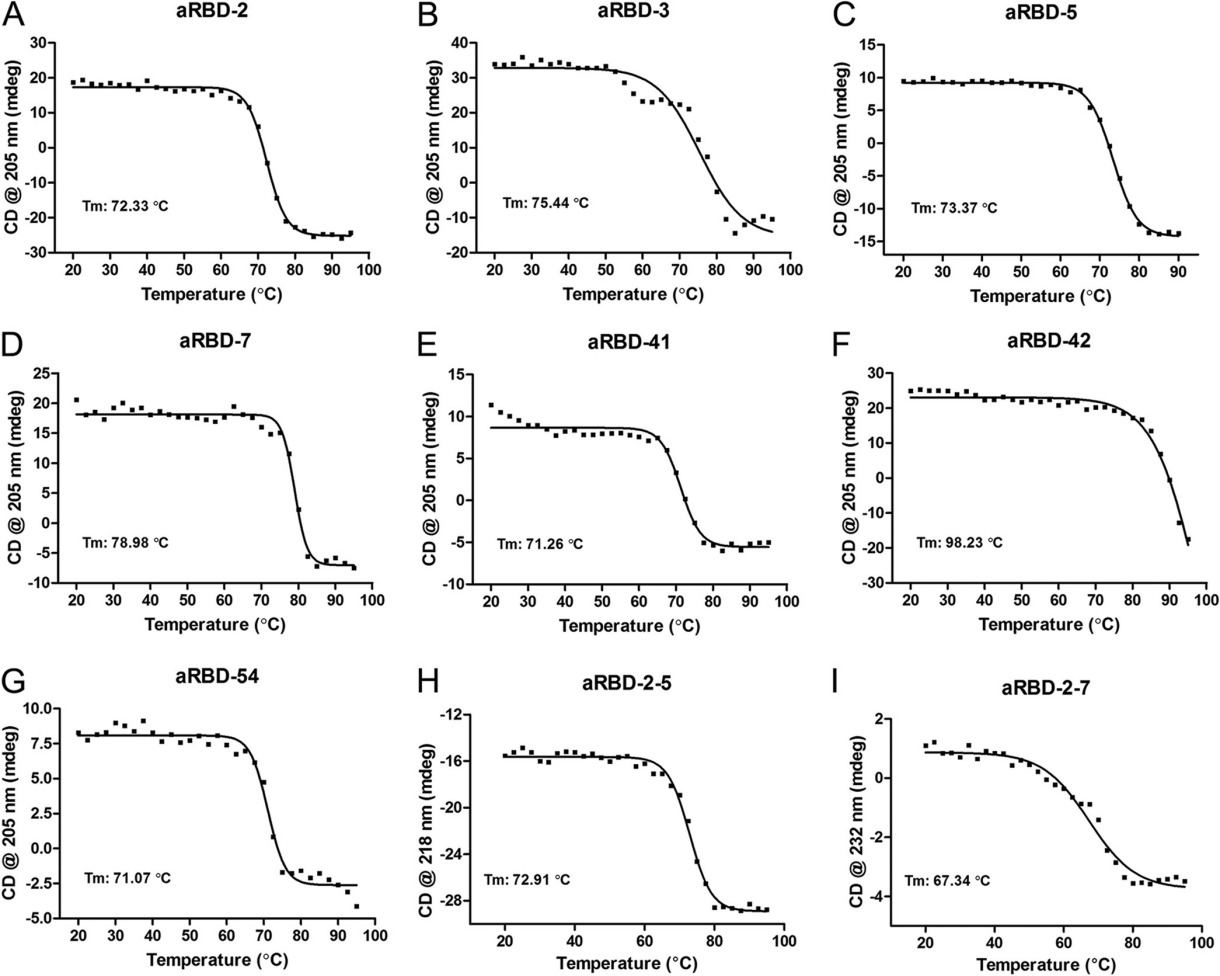
Thermal denature of Nbs by CD spectrum. (A to I) Thermal denature curves for aRBD-2, aRBD-3, aRBD-5, aRBD-7, aRBD-41, aRBD-42, aRBD-54, aRBD-2-5, and aRBD-2-7, respectively. Each experiment was repeated twice, the results data were fitted by Prism software.

The identified Nbs were expressed with a mammalian expression vector in 293F cells. To configure the Nb into an IgG-like molecule, we fused the C terminus of the identified Nbs to a tobacco etch virus (TEV) protease cleavage site and a human IgG1 Fc in a mammalian expression vector. The homo-bivalent Nb-TEV-Fc fusions were purified from the culture supernatant using protein A (Fig. 2D). All Nb-TEV-Fc fusions showed >100-mg/liter yields after 3 days of expression (data not shown). To prepare Nb monomers without the Fc, the fusion proteins were digested with the TEV protease (6×His tagged) and passed through protein G and a Ni-nitrilotriacetic acid (NTA) column. Highly purified Nbs were obtained from the flowthrough (Fig. 2E). The conformational stability of the seven Nbs was tested using circular dichroism, and the results showed that they were highly stable in solution, with the melting temperature exceeding 70°C (Fig. 3A to G).

### The identified nanobodies bind to RBD and spike ectodomain with nM affinities.

**FIG 4:**
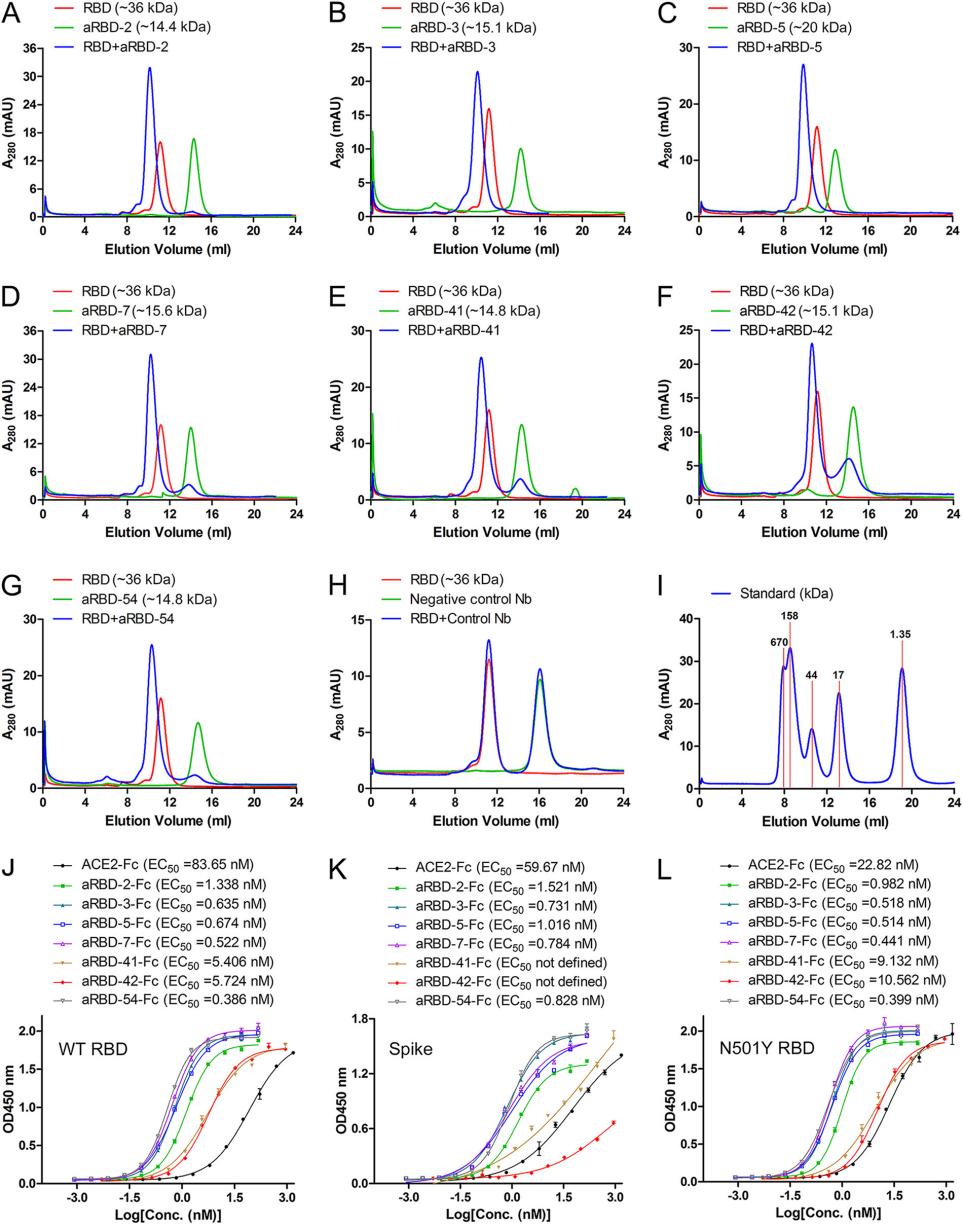
Interaction between Nbs and SARS-CoV-2 spike RBD or ectodomain in solution as measured by SEC and ELISA. (A to H). SARS-CoV-2 RBD, Nbs, and their 1:1 molar mixture were loaded over a Superdex 75 column (GE Healthcare). The elution peak from the mixtures came out earlier than those for RBD and Nbs. (I) Curve of standard. The binding affinities of the purified Nb-Fc fusions to SARS-CoV-2 spike RBD (J), the entire ectodomain (K), and the N501Y mutant RBD (L) were measured by ELISA. The EC_50_ was calculated by fitting the OD_450_ from serially diluted antibody with a sigmoidal dose-response curve. Error bars indicate means ± the standard deviations (SD) from two independent experiments.

The SARS-CoV-2 RBD-binding abilities of the seven Nbs were first verified using size exclusion chromatography (SEC). All seven Nbs formed stable complexes with RBD in solution (Fig. 4A to I). Furthermore, most Nb-Fc fusions demonstrated strong binding to both RBD and the entire ectodomain (S1+S2) of SARS-CoV-2 spike in ELISA, with a low nanomolar 50% effective concentration (EC_50_) (Fig. 4J and K). Compared to the human ACE2-Fc recombinant protein, these fusions bind to the RBD with a higher affinity (Fig. 4J), while all but aRBD-42 bind to the entire ectodomain of spike protein with a higher affinity (Fig. 4K). A N501Y point mutation was found in a SARS-CoV-2 variant that was rapidly spreading in the United Kingdom (31). As expected, the N501Y variant showed an enhanced binding activity with ACE2-Fc than the original RBD. Interestingly, the N501Y variant did not affect the binding to the seven Nbs (Fig. 4L).

**FIG 5:**
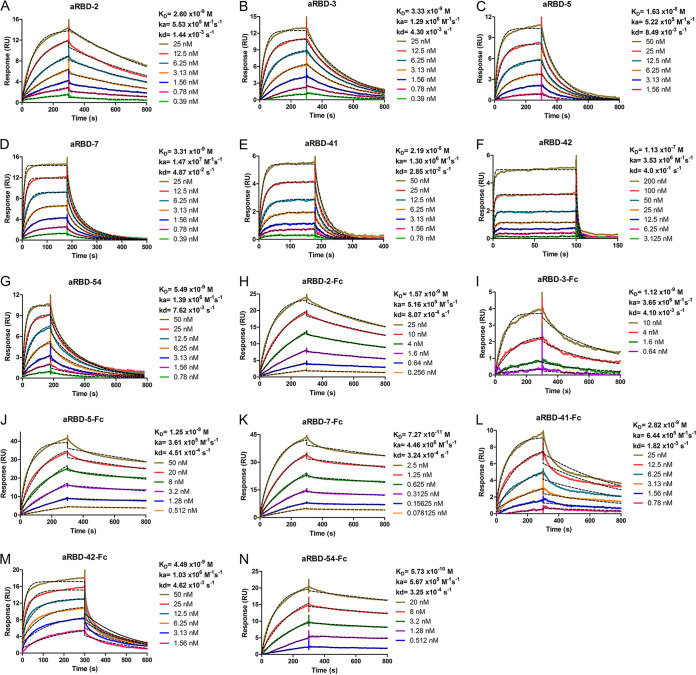
Characterization of binding affinity of isolated Nbs using SPR. The binding kinetics of aRBD-2 (A), aRBD-3 (B), aRBD-5 (C), aRBD-7 (D), aRBD-41 (E), aRBD-42 (F), aRBD-54 (G), and Nb-Fc fusions (H to N) were measured by SPR. The SARS-CoV-2 RBD was immobilized onto a CM5 sensor chip. Nbs and Nb-Fc fusions with serial 1:1 dilutions were injected and monitored by the Biacore T200 system. The actual responses (colored lines) and the data fitted to a 1:1 binding model (black dotted lines) are shown.

The binding affinity of the Nbs to RBD was also measured using surface plasmon resonance (SPR). Six Nbs showed a high binding affinity, with *K_D_* values of 2.60, 3.33, 16.3, 3.31, 21.9, and 5.49 nM for aRBD-2, aRBD-3, aRBD-5, aRBD-7, aRBD-41, and aRBD-54, respectively (Fig. 5A to E and G). Consistent with ELISA results, aRBD-42 had a relatively weaker binding affinity with a *K_D_* of 113 nM (Fig. 5F). Nb-Fc fusions showed an enhanced binding capability, with *K_D_* values ranging from 1.59 nM to 72.7 pM (Fig. 5H to N), probably due to an increase in avidity.

### Binding of the nanobodies to RBD mutants.

**FIG 6:**
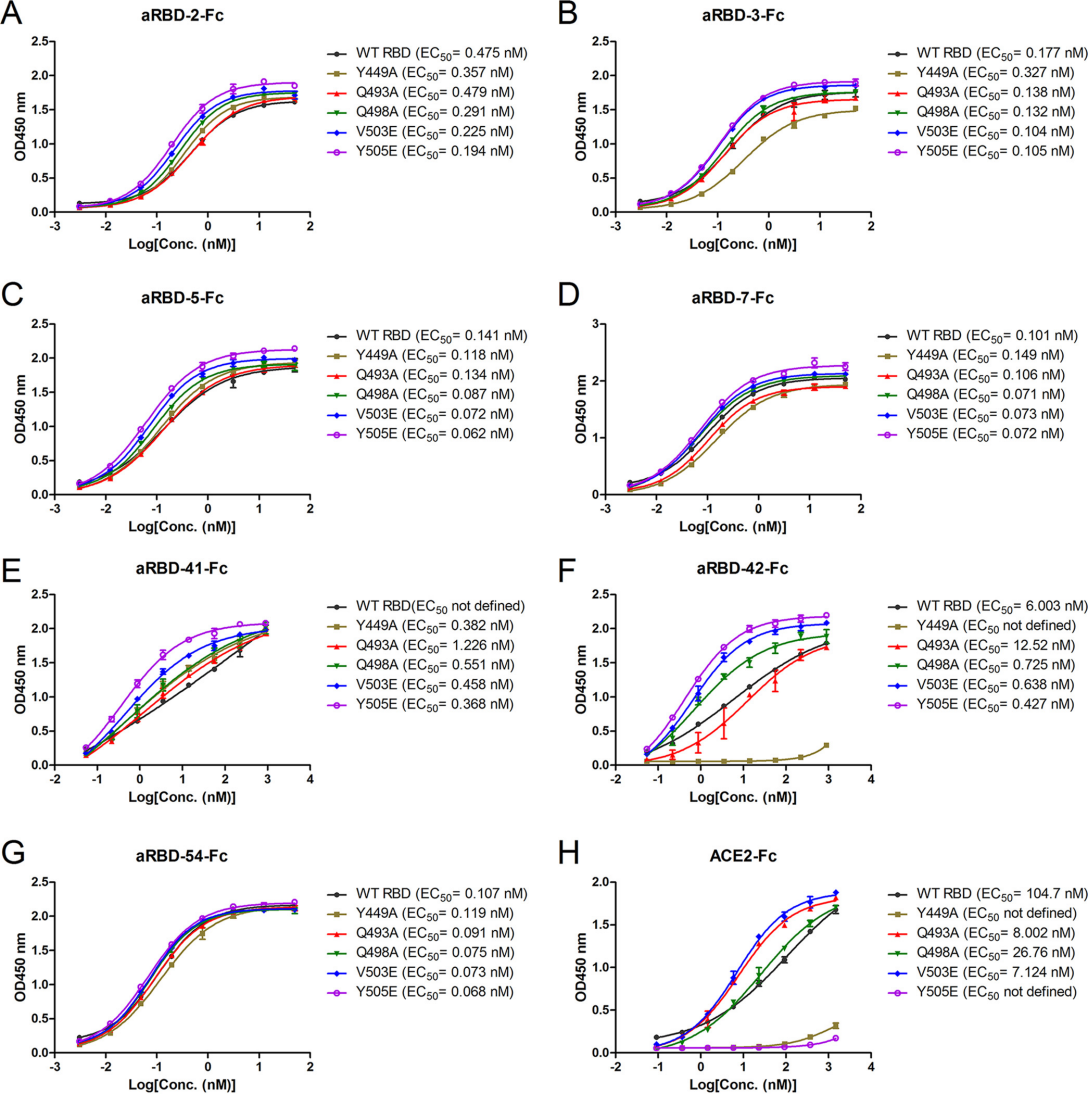
ELISA results for the characterization of identified Nbs and ACE2 binding to five RBD variants. (A to G) Results for aRBD-2-Fc, aRBD-3-Fc, aRBD-5-Fc, aRBD-7-Fc, aRBD-41-Fc, aRBD-42-Fc, and aRBD-54-Fc binding to the five RBD variants, respectively. (H) Results of ACE2 binding to the five RBD variants. The EC_50_ was calculated by fitting the OD_450_ from serially diluted antibody with a sigmoidal dose-response curve. Error bars indicate means ± the SD from two independent experiments.

In an effort to probe where the Nbs bind on RBD, we tested the effect of point mutations of RBD on binding with the Nbs. Five residues of RBD at the ACE2 binding interface (32) were chosen, and the recombinant RBD proteins with the individual mutation (Y449A, Q493A, Q498A, V503E, and Y505E) were purified (Fig. 1). Y449A and Y505E mutations weakened the binding to ACE2 (Fig. 6H), while the Q493A and V503E mutations enhanced their binding to ACE2 (Fig. 6H). In contrast, except for the Y449A mutation that greatly weakened the binding of aRBD-42, the other four mutations did not affect the binding with the other Nbs (Fig. 6A to G).

### Nbs block RBD-ACE2 interaction.

**FIG 7:**
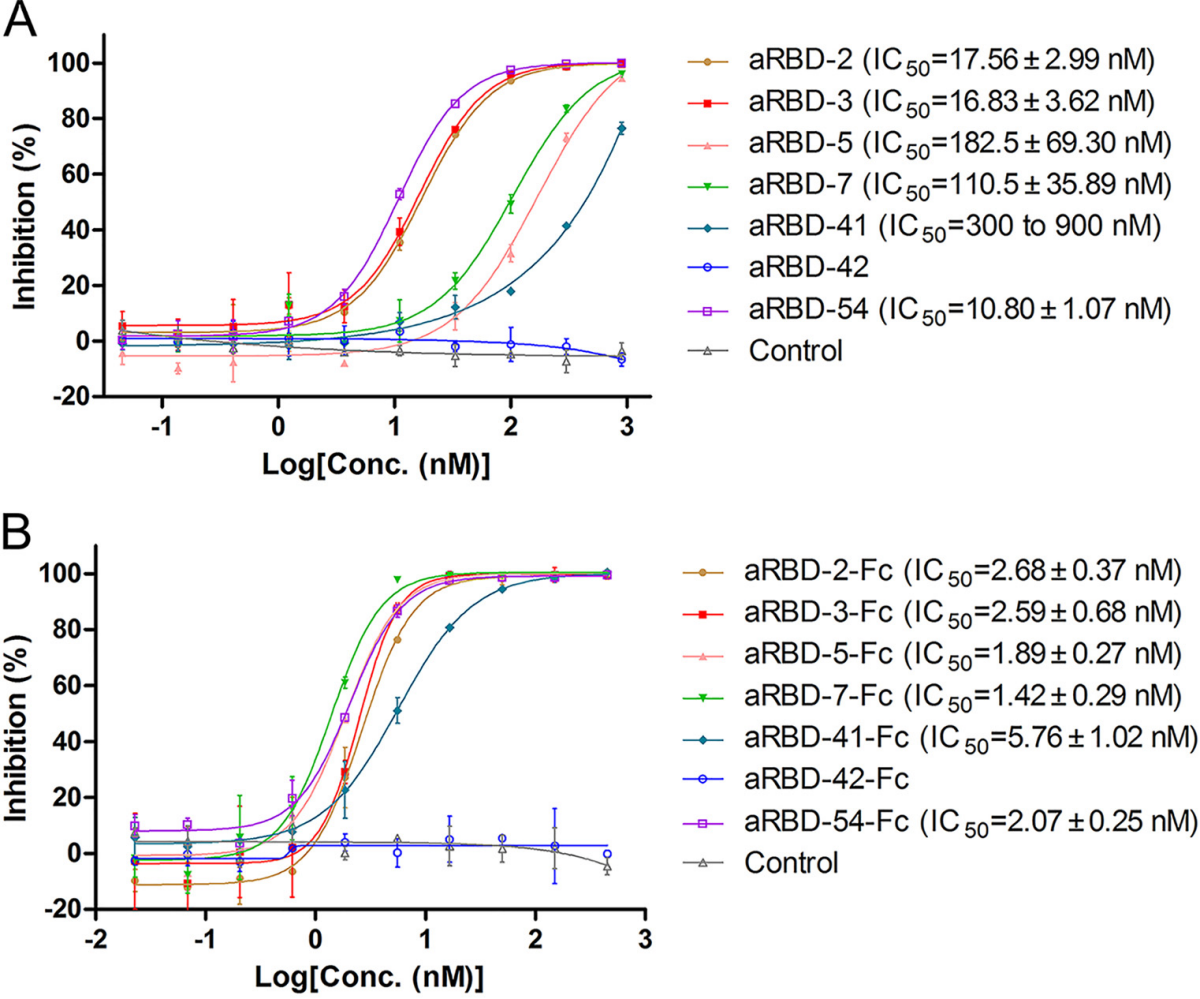
RBD-ACE2 blocking activities of isolated Nbs and their Fc fusions characterized by competitive ELISA. (A and B) Competitive ELISA of ACE-Fc binding to SARS-CoV-2 RBD immobilized on the plates by increasing concentrations of Nbs (A) or Nb-Fc fusions (B). After the competition, bound ACE2-Fc (A) or biotinylated ACE2-Fc (B) was detected by HRP-anti-IgG1 Fc antibody or streptavidin-HRP, respectively. Error bars indicate means ± the SD from two independent experiments. The IC_50_ was calculated by fitting the inhibition from serially diluted antibody to a sigmoidal dose-response curve.

The interaction of RBD and ACE2 initiates SARS-CoV-2 infection. To assess the ability of the Nbs in blocking RBD-ACE2 interaction, we performed competitive ELISA. Except for aRBD-42, which has the lowest RBD-binding affinity, all other Nbs (Fig. 7A) and their Fc fusions (Fig. 7B) effectively blocked the binding of ACE2-Fc and RBD in a dose-dependent manner. Compared to monovalent Nbs, Nb-Fc fusions showed enhanced blocking activities with a 5- to 90-fold decrease in half-maximal inhibitory concentration (IC_50_). The Nb-Fc fusions inhibited the binding of 10 nM ACE2-Fc to RBD with IC_50_ values at a nanomolar level, consistent with their binding affinities.

### High-affinity hetero-bivalent antibodies constructed based on epitope grouping.

**FIG 8:**
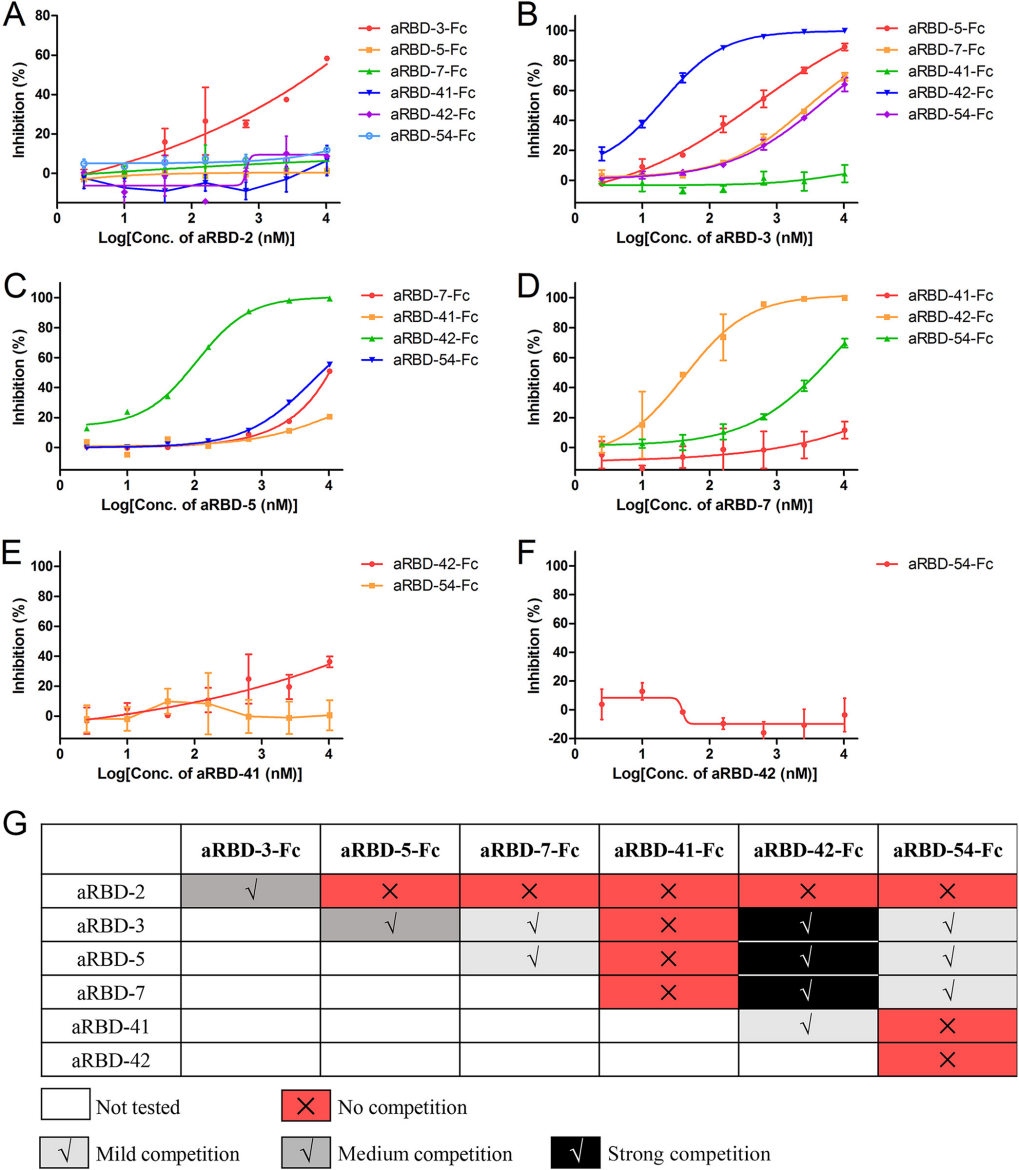
Epitope grouping results of the seven identified nanobodies. aRBD-2 (A), aRBD-3 (B), aRBD-5 (C), aRBD-7 (D), aRBD-41 (E), and aRBD-42 (F) were competed with other Nb-Fc fusions to bind SARS-CoV-2 RBD immobilized on the plates. The competition was determined by the reduction of the HRP-anti-IgG1 Fc induced chemiluminescence signal (OD_450_). The inhibition was calculated by comparing it to the Nb negative-control well. Error bars indicate means ± the SD from two independent experiments. (G) Competition strength is negatively correlated with OD_450_ signal, and the strength was summarized. aRBD-2 and aRBD-41 only showed moderate and mild competition with aRBD-3 and aRBD-42 for RBD binding, respectively. aRBD-3, aRBD-5, aRBD-7, and aRBD-42 showed mild to strong competition with each other for RBD binding. aRBD-3, aRBD-5, aRBD-7, and aRBD-54 also showed mild to strong competition with each other for RBD binding. aRBD-42 had no competition with aRBD-54.

To find out whether the Nbs bind to overlapping epitopes, the ability of the Nbs to compete with each other for RBD binding was studied with ELISA. The Nbs were serially diluted (ranging from 2.5 to 10,240 nM) and used to compete with 5 nM of Nb-Fc fusion to bind SARS-CoV-2 RBD coated on plates (Fig. 8A to F). The competition was summarized in Fig. 8G.

**FIG 9:**
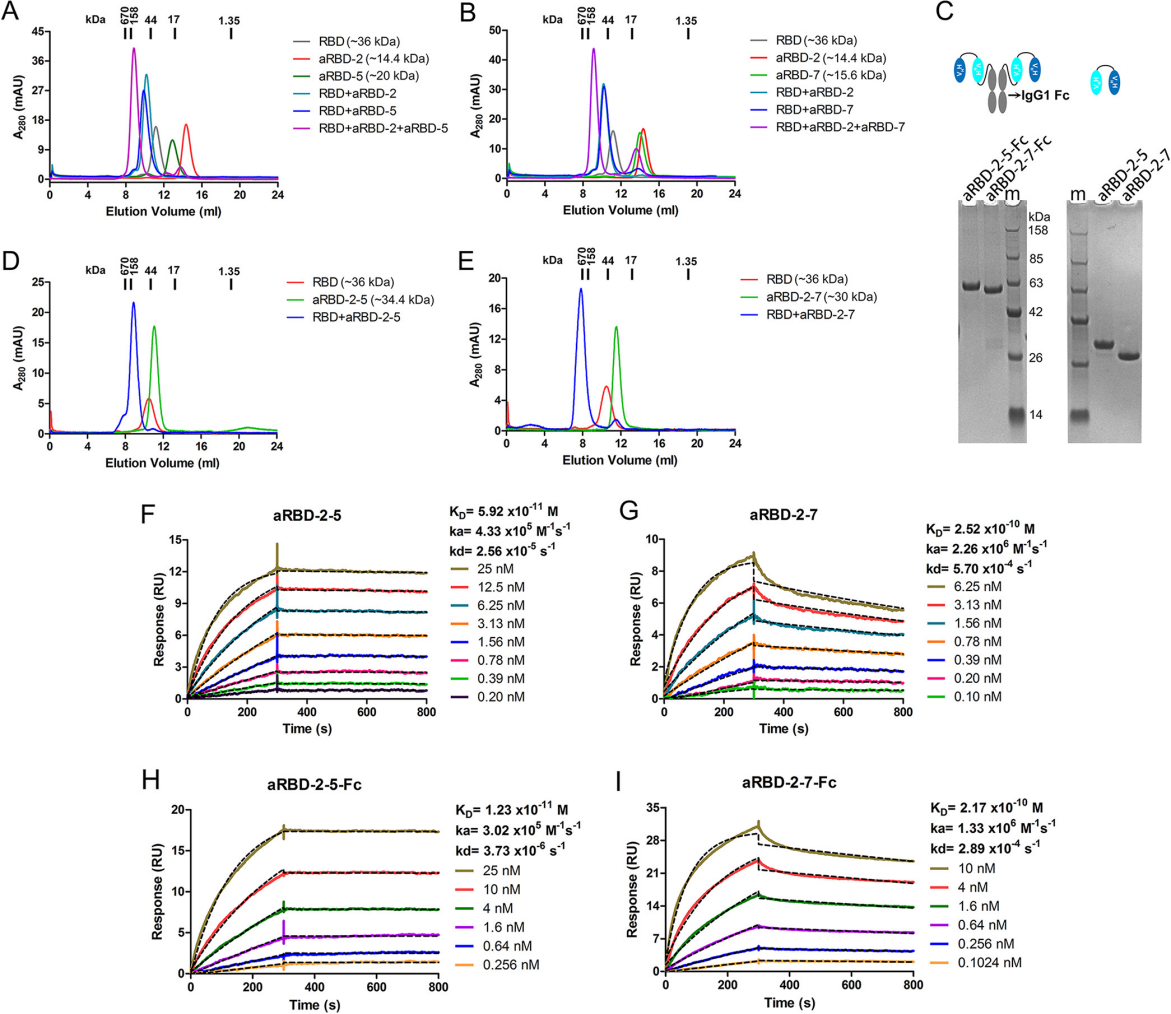
Preparation and characterization of the hetero-bivalent Nbs. (A) SEC profiling of RBD, aRBD-2, and aRBD-5 complex. (B) SEC profiling of RBD, aRBD-2, and aRBD-7 complex. (C) Reduced SDS-PAGE results of purified aRBD-2-5, aRBD-2-7, and their Fc fusions. (D) SEC profiling of RBD and aRBD-2-5 complex. (E) SEC profiling of RBD and aRBD-2-7 complex. The SARS-CoV-2 RBD binding kinetics of aRBD-2-5 (F), aRBD-2-7 (G), aRBD-2-5-Fc (H), and aRBD-2-7-Fc (I) were measured by SPR, respectively. The two hetero-bivalent Nbs with serially 1:1 dilutions were injected and monitored by using the Biacore T200 system. The actual responses (colored lines) and the data fitted to a 1:1 binding model (black dotted lines) are shown.

Based on these grouping and additional SEC results (Fig. 9A and B), we engineered two hetero-bivalent Nbs, namely, aRBD-2-5 and aRBD-2-7, by connecting aRBD-2 head-to-tail with aRBD-5 and aRBD-7 through a (GGGGS)_3_ flexible linker, respectively. They were also expressed in 293F cells and purified (Fig. 9C). SEC indicated aRBD-2-5 and aRBD-2-7 were monomeric in solution (Fig. 9D and E), and circular dichroism spectrum analysis showed they were also highly stable in solution (Fig. 3H and I). The RBD binding activities of aRBD-2-5 and aRBD-2-7 were studied using SEC (Fig. 9D and E) and SPR. In contrast to the monovalent Nbs, the hetero-bivalent aRBD-2-5 and aRBD-2-7 showed greatly enhanced binding affinities, with *K_D_* values of 59.2 pM and 0.25 nM, respectively (Fig. 9F and G). Similarly, their Fc fusions also showed enhanced binding affinities, with *K_D_* values of 12.3 pM and 0.22 nM, respectively (Fig. 9H and I).

### Hetero-bivalent Nbs exhibit potent neutralizing ability against live SARS-CoV-2.

**FIG 10:**
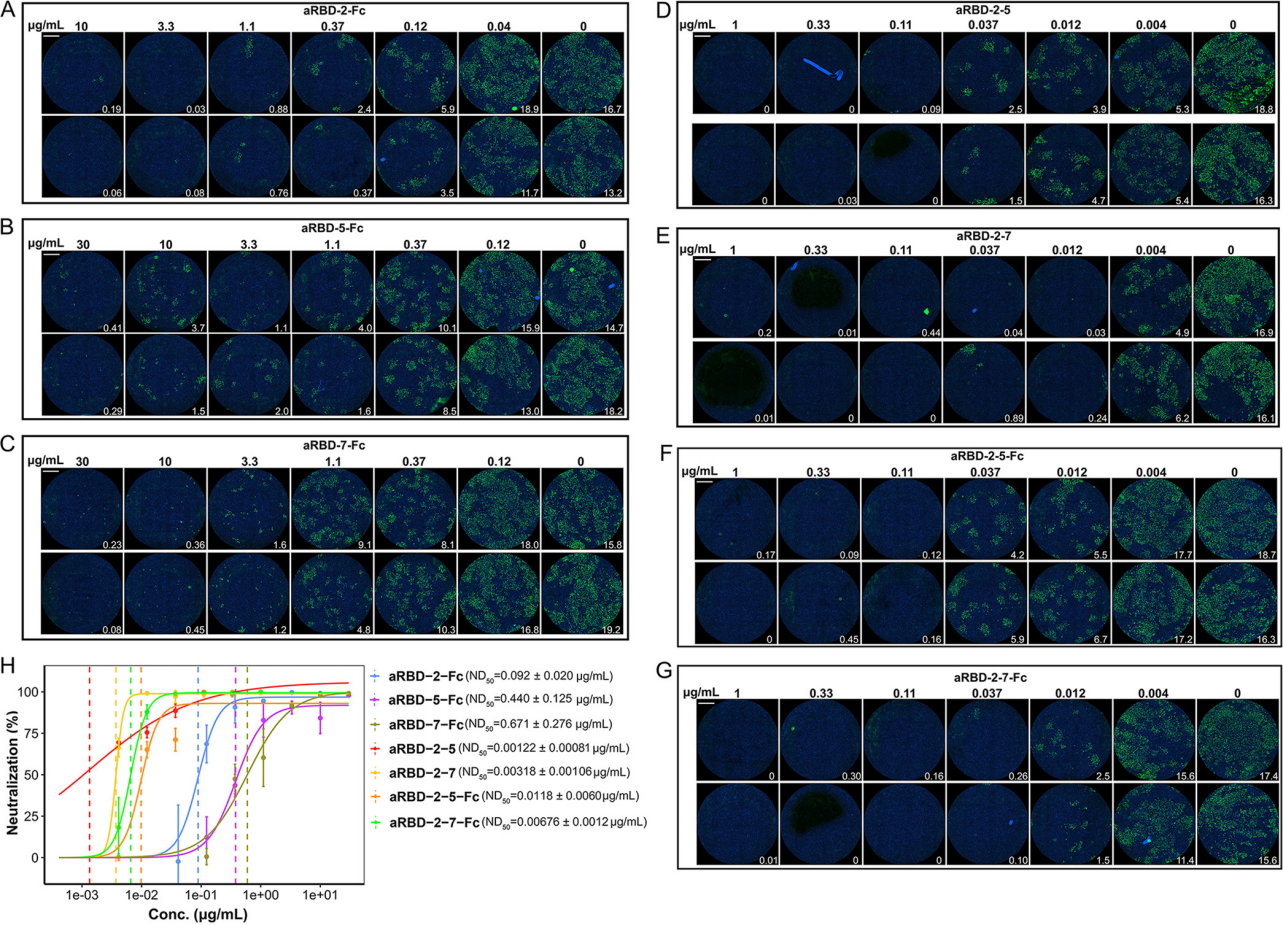
*In vitro* SARS-CoV-2 neutralization of the homodimeric Nbs, hetero-bivalent Nbs, and their Fc fusions. Serially diluted aRBD-2-Fc (A), aRBD-5-Fc (B), aRBD-7-Fc (C), aRBD-2-5 (D), aRBD-2-7 (E), aRBD-2-5-Fc (F), and aRBD-2-7-Fc (G) were incubated with ∼200 PFU of SARS-CoV-2. The mixture was then added to Vero E6 cells in 96-well plates. After 2 days of infection, the infected virus was stained green with a monoclonal antibody against SARS-CoV-2 NP and an Alexa Fluor 488-conjugated goat anti-mouse secondary antibody. The nucleus was stained blue with Hoechst 33342. Each experiment was performed in duplicate (the upper and lower images). An immunofluorescence image of the entire well is shown with the numbers in the low right corner representing the percentage of cells that are NP positive. (H) ND_50_s of the identified Nbs and their Fc fusions were calculated by fitting the data in panels A to G with a log-logistic model (45). The data are fitted to the model with the drc package in R to obtain the 95% confidence intervals. Error bars indicate means ± the SD from two independent experiments.

To assess the ability of the Nbs in neutralizing SARS-CoV-2, we developed a SARS-CoV-2 microneutralization assay and assessed representative Nbs in this assay. Nbs serially diluted to different concentrations were incubated with ∼200 PFU of SARS-CoV-2 and inoculated onto Vero E6 cells in 96-well plates. The inoculum was removed after 1 h, and the cells were covered with a semisolid medium for 2 days before the infection was assessed by an immunofluorescence assay utilizing antibodies specific for SARS-CoV-2 N protein. Three representative monomeric Nbs—aRBD-2, aRBD-5, and aRBD-7—showed only a modest level of neutralization at antibody concentrations of 33 to 100 μg/ml (Table 1). aRBD-2 was more effective than aRBD-5 and aRBD-7 in neutralizing SARS-CoV-2 (data not shown), correlating with its higher binding affinity to RBD. In contrast, the dimeric Nbs showed greatly enhanced neutralizing potency. The homo-bivalent aRBD-2-Fc, aRBD-5-Fc, and aRBD-7-Fc exhibited 50% neutralization doses (ND_50_s) of 0.092 μg/ml (∼1.12 nM), 0.440 μg/ml (∼5.34 nM), and 0.671 μg/ml (∼8.02 nM), respectively (Fig. 10A to C and H), again correlating with their RBD binding affinities. Interestingly, the hetero-bivalent Nbs exhibited an even higher neutralizing potency than the homodimeric Nbs. The fitted ND_50_s for aRBD-2-5 and aRBD-2-7 are 1.22 ng/ml (∼0.043 nM) and 3.18 ng/ml (∼0.111 nM), respectively (Fig. 10D, E and H). The Fc fusions of the hetero-bivalent Nbs did not further increase the neutralization potency. The ND_50_s for aRBD-2-5-Fc and aRBD-2-7-Fc are 11.8 ng/ml (∼0.107 nM) and 6.76 ng/ml (∼0.0606 nM), respectively (Fig. 10F, G, and H).

**TABLE 1:** RBD binding affinity *K_D_* and ND50 of neutralizing 200 PFU of SARS-CoV-2

Nb or Nb construct	Affinity *K_D_* (nM)	ND_50_ (μg/ml)	ND_50_ (nM)
aRBD-2	2.60	33–100	2,000–6,600
aRBD-3	3.33	NT^*a*^	NT
aRBD-5	16.3	33–100	2,000–6,600
aRBD-7	3.31	33–100	2,000–6,600
aRBD-41	21.9	NT	NT
aRBD-42	113	NT	NT
aRBD-54	5.49	NT	NT
aRBD-2-Fc	1.57	0.092	1.12
aRBD-3-Fc	1.12	NT	NT
aRBD-5-Fc	1.25	0.440	5.34
aRBD-7-Fc	0.073	0.671	8.02
aRBD-41-Fc	2.82	NT	NT
aRBD-42-Fc	4.49	NT	NT
aRBD-54-Fc	0.573	NT	NT
aRBD-2-5	0.059	0.00122	0.043
aRBD-2-7	0.252	0.00318	0.111
aRBD-2-5-Fc	0.012	0.0118	0.107
aRBD-2-7-Fc	0.217	0.00676	0.061

a Nb, nanobody; ND_50_, 50% neutralization dose; NT, not tested.

## DISCUSSION

The infection of epithelial cells by SARS-CoV-2 is initiated by the interaction between the Spike RBD and ACE2 (25, 33). Hence, RBD-targeting antibodies hold promise as prophylactics and therapeutics for SARS-CoV-2. Here, seven unique Nbs were isolated from RBD-immunized alpacas, and amino acid sequence alignment shows CDR sequences of these Nbs are distinct from other reported Nbs (34–42). Four of the Nbs exhibited a high affinity of low-nanomolar *K_D_* (Fig. 5), similar to recently reported natural (34, 35) and synthetic Nbs (36–38). We also measured the RBD-binding affinity of the Nb-Fc fusions. Due to the bivalent nature and ∼6-fold increase in the molecular weight, most of the Nb-Fc chimeric antibodies showed a higher affinity (*K_D_* ranging from 72.7 pM to 4.5 nM) than their monomeric counterparts (Fig. 5). This affinity is even higher than that of some MAbs isolated from lymphocytes of convalescent COVID-19 patients (16–18).

Except for the Nb with the lowest affinity for RBD (aRBD-42; *K_D_* of 113 nM), the other six Nbs can block the interaction between ACE2 and RBD. aRBD-2, aRBD-3, and aRBD-54, which had higher RBD-binding affinities, showed a stronger ACE-RBD blocking capacity than aRBD-5 and aRBD-41 (Fig. 7A). However, aRBD-7, which had a similarly high RBD binding affinity of 3.31 nM, only exhibited a weak ACE2-RBD blocking activity (Fig. 7A). We thus infer that different Nbs may occupy different epitopes on RBD, leading to the various strengths of ACE2 binding interference. The epitopes of some Nbs may overlap more closely with that of ACE2. Interestingly, even when the Nbs with a relatively weak ACE2-RBD blocking ability were fused with IgG1 Fc to form homodimers, their blocking ability was increased >75-fold (Fig. 7A and B). This effect is probably due to the increased apparent RBD-binding affinity by dimerization and the additional steric hindrance caused by the increased size. Further investigations are needed to understand the underlying mechanisms.

According to grouping results of the seven Nbs, two hetero-bivalent antibodies were constructed by fusing aRBD-2 to aRBD-5 and aRBD-7 tail-to-head with a flexible linker, which achieved a >10-fold increase in RBD-binding affinity (Fig. 9F and G). Consistent with the increased affinity and steric hindrance, the SARS-CoV-2 neutralization potencies of aRBD-2-5 and aRBD-2-7 were greatly enhanced, with ND_50_s of 1.22 ng/ml (∼0.043 nM) and 3.18 ng/ml (∼0.111 nM) (Fig. 10). Table 1 summarizes the binding and neutralization data for each nanobody or construct. Besides, compared to the wild-type RBD, the RBD containing N501Y mutation showed a similar binding ability to the isolated Nbs (Fig. 4J and L), indicating that our hetero-bivalent Nbs are likely to be applicable to neutralize the new SARS-CoV-2 variant that is rapidly spreading in the United Kingdom.

In summary, we identified several high-affinity natural Nbs with RBD-ACE2 blocking ability and two hetero-bivalent Nbs with potent SARS-CoV-2 neutralization capacity. Alpaca V_H_H has a high degree of homology with human V_H_3, so it has low immunogenicity in humans (43, 44). These Nbs can be further improved concerning their antiviral function through affinity maturation or genetic modification, potentially serving as therapeutics for treating COVID-19.

## MATERIALS AND METHODS

### Protein expression and purification.

The coding sequences for SARS-CoV-2 RBD (amino acids [aa] 321 to 591), SARS-CoV-2 RBD variants, SARS-CoV-2 S1 (aa 1 to 681), SARS-CoV-1 RBD (aa 309 to 540), human ACE2 extracellular domain (aa 19 to 615), and the identified Nbs were appended with a TEV enzyme site and a human IgG1 Fc at the C terminus, as well as the IFNA1 signal peptide at the N terminus. The fusions were cloned into the mammalian expression vector pTT5. The expression vectors were transiently transfected to human HEK293F cells with polyethyleneimine (Polyscience). Three days later, cell supernatants were obtained by centrifugation at 3,000 × *g* for 10 min, diluted 1:1 with the running buffer (20 mM Na_2_HPO_4_, 150 mM NaCl [pH 7.0]), and loaded on a protein A column. The bound protein was eluted with 100 mM acetic acid on ÄKTA pure (GE Healthcare). To remove IgG1 Fc, the purified fusion proteins were first digested with the 6×His-tagged TEV enzyme. Protein A (protein G for Nbs) and Ni-NTA were then used sequentially to remove the undigested fusion protein, Fc, and the TEV enzyme. Fc-free recombinant proteins were collected from the flowthrough. The protein purity was estimated by SDS-PAGE (Fig. 1), and the concentration was measured by using a spectrophotometer (Analytik Jena).

### Phage display library construction.

The experiments involved alpacas were approved by a local ethics committee. Two female alpacas were immunized twice by subcutaneous injection and once by intramuscular injection, each with 500 μg of SARS-CoV-2 RBD in phosphate-buffered saline (PBS), emulsified with an equal volume of Freund adjuvant (Sigma-Aldrich). At 2 weeks after the final boost, more than 1 × 10^7^ lymphocytes were isolated from peripheral blood by Ficoll 1.077 (Sigma-Aldrich) separation, and the total RNA from the lymphocytes was isolated using a total RNA kit (Omega Bio-Tek) according to the manufacturer’s protocol. First-strand cDNA synthesis was performed with 4 μg of total RNA per reaction by using a PrimeScript II first-strand cDNA synthesis kit and oligo(dT) primer (TaKaRa) according to the manufacturer’s protocol. The variable domain of heavy-chain only antibody (V_H_H) was amplified by PCR with the forward primer GCTGCACAGCCTGCTATGGCACAGKTGCAGCTCGTGGAGTCTGGGGG and the reverse primer GAGTTTTTGTTCGGCTGCTGCTGAGGAGACGGTGACCTGGGTCCCC. The phagemid pR2 was amplified by PCR with the forward primer AGCAGCCGAACAAAAACTCATCTCAGAAGAG and the reverse primer CCATAGCAGGCTGTGCAGCATAGAAAGGTACCACTAAAGGAATTGC. Next, 2 pmol of the V_H_H fragments and 0.5 pmol of the amplified pR2 vector were mixed and diluted to 50 μl. An equal volume of 2× Gibson Assembly mix was added to the mixture, followed by incubation at 50°C for 1 h. The ligation was cleaned up by using a Cycle Pure kit (Omega Bio-Tek) and transformed into TG1 electrocompetent cells in a 0.1-cm electroporation cuvette using the BTX ECM 399 electroporation system (Harvard Apparatus) with the following settings: 2.5 kV and 5 ms. The transformants were spread on five 150-mm TYE agar plates supplemented with 2% glucose and 100 μg/ml ampicillin, followed by overnight culture at 37°C. The colonies were scraped from the plates with a total of 20 ml 2×TY medium and thoroughly mixed. Then, 200 μl of the liquid was inoculated into 200 ml of 2×TY to amplify the library. Phage particles displaying V_H_H were rescued from the library using KM13 helper phage.

### Biopanning and selection of positive clones.

Two rounds of panning were performed. Immuno MaxiSorb plates (Nunc) were coated with 0.1 ml of SARS-CoV-2 RBD solution (100 and 20 μg/ml in the first and second rounds, respectively). Control wells without antigen coating were used in parallel in every round of panning. After blocking with MPBS (i.e., PBS supplemented with 5% milk powder) for 2 h at room temperature, 1 × 10^11^ PFU of the library phages were added for the first round of selection. The wells were washed with PBST (PBS supplemented with 0.1% Tween 20) 20 times to remove the unbound phages. Bound phages were eluted by digestion with 100 μl of 0.5 mg/ml trypsin for 1 h at room temperature. The eluted phages were used to infect Escherichia coli TG1 for titer determination and amplification. The second round of panning was performed similarly with the following differences: the amount of input phage was 1 × 10^8^ PFU, the washing time was 30 times, and the concentration of Tween 20 in washing buffer was 0.2%.

A total of 31 individual clones from each round of panning were picked and identified using monoclonal phage ELISA. The monoclonal phage was rescued with helper phage KM13 and added to the well coated with 0.1 μg of RBD. After 1 h of incubation at room temperature, the wells were washed four times with PBST, and HRP-anti-M13 antibody was added. After four washes with PBST, TMB (Beyotime) was added to each well, followed by incubation in the dark at room temperature for 2 min. The chromogenic reaction was stopped with 50 μl of 1 M sulfuric acid, and the optical density at 450 nm (OD_450_) was determined. The clone with an OD_450_ that was 20 times higher than that of the control well was defined as a positive clone. The phagemids extracted from the positive clones were sequenced.

### Size-exclusion chromatography.

The interaction of SARS-CoV-2 RBD and the Nbs in solution was studied by using gel filtration. SARS-CoV-2 RBD, Nbs, and their mixture (1.6 nmol of SARS-CoV-2 RBD mixed with 1.6 nmol of Nbs) were run over a Superdex 75 column (GE Healthcare) at a flow rate of 0.5 ml/min with AKTA pure.

### ELISA.

Immuno-MaxiSorb plates (Nunc) were coated and blocked as described above. For noncompetitive ELISA of purified Nb-Fc and ACE2-Fc binding assay, Nb-Fc and ACE2-Fc solutions serially diluted 1:3 were added to the plates, followed by incubation for 1 h at room temperature. After four washes with PBST, bound Nb-Fc and ACE2-Fc were detected with a monoclonal anti-IgG1 Fc-HRP antibody (Sino Biological). To characterize the epitope competition between the identified Nbs, serially 1:4 diluted Nb solutions (ranging from 2.5 to 10,240 nM) were mixed with 5 nM Nb-Fc solutions. After incubation in RBD-coated wells and standard washing, bound, Nb-Fc was detected with a monoclonal anti-IgG1 Fc-HRP antibody. For the ACE2-RBD blocking assay, serially 1:3 diluted Nb solutions (ranging from 0.046 to 900 nM) and Nb-Fc solution (ranging from 0.023 to 450 nM) were mixed with 10 nM ACE2-Fc and 10 nM biotinylated ACE2-Fc, respectively. After incubation in RBD coated wells and standard washing, bound ACE2-Fc and biotinylated ACE2-Fc was detected with an anti-IgG1 Fc-HRP antibody or HRP-streptavidin, respectively. The chromogenic reaction and the OD_450_ measurement were similarly performed as for phage ELISA.

### Circular dichroism.

Secondary structure and thermal stabilities of identified Nbs were studied by circular dichroism (CD) spectra using a Chirascan spectrometer (Applied Photophysics). Prior to CD measurements, the sample buffer was changed to PBS, and the protein concentration was adjusted to 0.3 mg/ml. CD spectra were acquired for each sample from 180 to 260 nm using a 1-mm path-length cell. For thermal titration, CD spectra were acquired between 20 and 95°C, with temperature steps of 2.5°C. CD signals at 205 nm were used to characterize the structural changes during thermal titration. Each experiment was repeated twice, and the data were fitted with Prism to obtain the *T_m_* values.

### Surface plasmon resonance.

SPR measurements were performed at 25°C using a BIAcore T200 system. SARS-CoV-2 RBD was diluted to a concentration of 15 μg/ml with sodium acetate (pH 4.5) and immobilized on a CM5 chip (GE Healthcare) at a level of ∼150 response units. All proteins were exchanged into the running buffer (PBS [pH 7.4] supplemented with 0.05% Tween 20), and the flow rate was 30 μl/min. The blank channel of the chip served as the negative control. For affinity measurements, a series of different concentrations of antibodies flowed over the sensorchip. After each cycle, the chip was regenerated with 50 mM NaOH buffer for 60 to 120 s. The sensorgrams were fitted with a 1:1 binding model using Biacore evaluation software.

### SARS-CoV-2 neutralization assay.

Nbs and Nb-Fc fusions in a 3-fold dilution concentration series were incubated with ∼200 PFU of SARS-CoV-2 (USA-WA1/2020 isolate) for 30 min. The antibody and virus mixture was then added to Vero E6 cells in 96-well plates (Corning). After 1 h, the supernatant was removed from the wells, and the cells were washed with PBS and overlaid with Dulbecco modified Eagle medium (DMEM) containing 0.5% methylcellulose. After 2 days of infection, the cells were fixed with 4% paraformaldehyde, permeabilized with 0.1% Triton-100, blocked with DMEM containing 10% fetal bovine serum, and stained with a rabbit monoclonal antibody against SARS-CoV-2 NP (GeneTex, GTX635679) and an Alexa Fluor 488-conjugated goat anti-mouse secondary antibody (Thermo Fisher Scientific). Hoechst 33342 was added in the final step to counterstain the nuclei. Fluorescence images of the entire well were acquired with a 4× objective in a Cytation 5 (BioTek). The total number of cells indicated by the nuclei staining and the infected cells indicated by the NP staining were quantified with the cellular analysis module of the Gen5 software (BioTek). All experiments involving live SARS-CoV-2 were carried out under BSL-3 containment. A log-logistic model (45) was used to model the dose-response curves of the antibodies. The data are fitted to the model with the drc package in R to obtain the 95% confidence intervals and ND_50_. All of these Nbs were lyophilized at a concentration of 2 to 5 mg/ml and kept at room temperature for 1 week for transportation. The lyophilized Nbs were redissolved in ddH_2_O in the neutralization assay.
